# Combining a GSI and BCL-2 inhibitor to overcome melanoma's resistance to current treatments

**DOI:** 10.18632/oncotarget.13141

**Published:** 2016-11-05

**Authors:** Nabanita Mukherjee, Adam Almeida, Katie A. Partyka, Yan Lu, Josianna V. Schwan, Karoline Lambert, Madison Rogers, William A Robinson, Steven E Robinson, Allison J Applegate, Carol M Amato, Yuchun Luo, Mayumi Fujita, David A. Norris, Yiqun G. Shellman

**Affiliations:** ^1^ University of Colorado Anschutz Medical Campus, School of Medicine, Department of Dermatology, Aurora, CO 80045, USA; ^2^ Division of Medical Oncology, University of Colorado Anschutz Medical Campus, School of Medicine, Aurora, CO 80045, USA; ^3^ Department of Veterans Affairs Medical Center, Dermatology Section, Denver, CO 80220, USA

**Keywords:** melanoma stem cells, melanoma initiating cells, GSI-I, ABT-737, BRAF-WT melanoma

## Abstract

Major limitations of current melanoma treatments are for instances of relapse and the lack of therapeutic options for BRAF wild-type patients who do not respond to immunotherapy. Many studies therefore focus on killing resistant subpopulations, such as Melanoma Initiating Cells (MICs) to prevent relapse. Here we examined whether combining a GSI (γ-Secretase Inhibitor) with ABT-737 (a small molecule BCL-2/BCL-XL/BCL-W inhibitor) can kill both the non-MICs (bulk of melanoma) and MICs. To address the limitations of melanoma therapies, we included multiple tumor samples of patients relapsed from current treatments, with a diverse genetic background (with or without the common BRAF, NRAS or NF1 mutations) in these studies. Excitingly, the combination treatment reduced cell viability and induced apoptosis of the non-MICs; disrupted primary spheres, decreased the ALDH+ cells, and inhibited the self-renewability of the MICs in multiple melanoma cell lines and relapsed patient samples. Using a low-cell-number mouse xenograft model, we demonstrated that the combination significantly reduced the tumor initiating ability of MIC-enriched cultures from relapsed patient samples. Mechanistic studies also indicate that cell death is NOXA-dependent. In summary, this combination may be a promising strategy to address treatment relapse and for triple wild-type patients who do not respond to immunotherapy.

## INTRODUCTION

Metastatic melanoma is one of the most devastating forms of skin cancer. It is the fifth most common cancer in men and the seventh in women with an estimated 76,380 new cases and 10,130 deaths for 2016 in the U.S. [1]. Common genes associated with aggressive melanoma include BRAF, with these mutations comprising the majority of melanoma cases (37–50%), followed by NRAS (13–25%) and NF1 (11.9%) [2]. BRAF-targeting drugs have provided a major breakthrough in melanoma treatment, but have limitations due to high relapse and increased resistance to therapy [3–6]. Moreover, BRAF wild type (WT) tumors do not respond to molecular-targeted treatments and immunotherapy is the main treatment option for these patients [6]. However, not all patients respond to immunotherapy and there are no known biomarkers to predict this response [6]. In addition, immunotherapy treatments may pose risks of immune-related side effects [7, 8]. Thus, in spite of the ground breaking progresses in melanoma treatment, relapse and lack of treatment options for BRAF-WT patients remains a crucial issue.

Research suggests that the relapse and resistance to therapy observed for melanoma and other cancers can be due to cancer stem cells (CSCs), a sub-population of cells within a tumor that can self-renew and give rise to tumor heterogeneity [9–11]. Thus, targeting CSCs has become an integral goal for cancer researchers [9–11]. Studies by independent labs provide evidence about the existence of a sub-population of melanoma cells possessing similar characteristics to other CSCs. These cells are termed melanoma initiating cells (MICs), characterized by their extensive self-renewal capacity, tumor initiation and propagation, and chemo-resistance [12–19]. Current therapies for melanoma do not account for MICs, and this may be one of the reasons for the high relapse rate. Thus, it is crucial to look for therapies that would eliminate not only the bulk of melanoma cells, but also MICs.

γ-Secretase Inhibitors (GSIs) have been found to target cancer stem cells in pre-clinical studies and have been tested or are in clinical trials for cancer treatment [20]. BCL-2 inhibitors have been successful in treatment of chronic lymphocytic leukemia [21, 22], but not in melanoma due to high expression of anti-apoptotic proteins such as MCL-1 [23–26]. In melanoma, the NOXA/MCL-1/BCL-2 apoptotic node has emerged as a common vulnerable spot for targeting the MIC [24, 27, 28] and non-MIC populations of melanoma [24– 30]. The induction of the pro-apoptotic protein NOXA selectively inhibits MCL-1 and is able to overcome apoptotic resistance. Our previous works suggested that the combination therapy of ABT-737 (a small molecule BCL-2, BCL- XL, and BCL-W inhibitor) with NOXA-inducing compounds can be an effective strategy to de-bulk and eliminate MICs [24, 27, 28]. GSI-I upregulates NOXA [31, 32], which makes it an attractive candidate to test in combination with ABT-737.

Another major drawback of current melanoma treatments is the lack of therapeutic options for patients who are WT for common mutations in BRAF, NRAS or NF-1 (Triple-WT) [33]. These patients often do not respond well to immunotherapy [34–37]. To be more clinically relevant to the landscape of current melanoma treatments, we extended our studies to melanoma tumor samples from patients with various genetic backgrounds and that were recently relapsed from current treatments. Samples included mutated BRAF/NRAS, or Triple-WT. Most of the patient tumor samples used here have relapsed from the molecular-targeted treatment, immunotherapy, or treatments of multiple chemotherapies and radiation. The aim of this study was to test the efficacy of GSI-I and ABT-737 to de-bulk and kill MICs in conventional cell lines as well as a genetically diverse population of relapsed patient samples. The results here suggest that the use of this combination is a promising treatment strategy for melanoma regardless of the mutation status of BRAF or NRAS, and it may overcome melanoma's resistance to current treatments.

## RESULTS

### GSI-I in combination with ABT-737 reduced cell viability and killed melanoma cells, but not normal melanocytes in monolayer conditions

**Figure 1:**
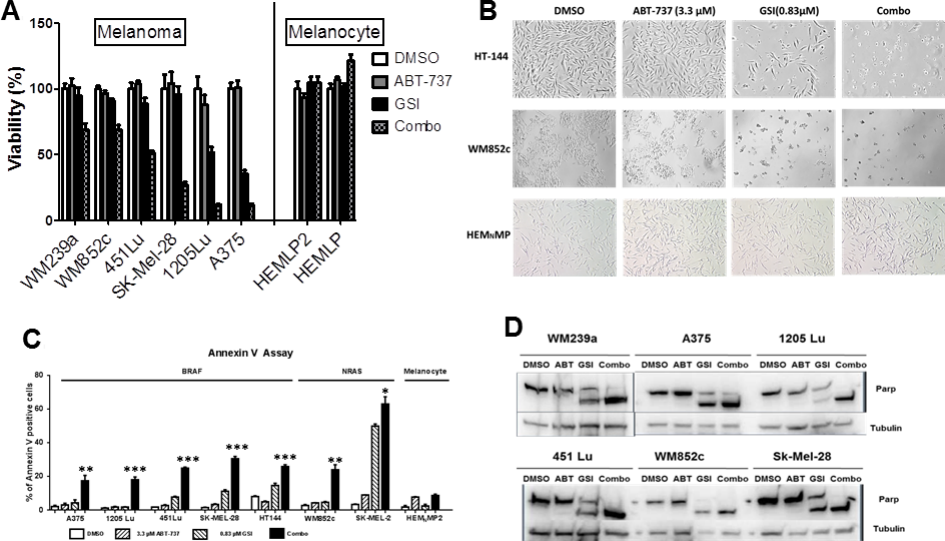
GSI-I combined with ABT-737 reduces cell viability and induces apoptosis in melanoma cells, but not normal melanocytes in monolayer culture conditions (**A**) MTS assays of six melanoma cell lines and two human primary melanocyte cultures post indicated treatments. The viability of the DMSO control for each cell line was set to 100%. The combination significantly (*p* < 0.05 or less) reduced cell viability compared with DMSO or with single drug treated conditions in all melanoma cell lines. The statistical information was not added as it will make the figure difficult to read. (**B**) Bright field analysis of the experiment in Figure 1A. Scale bar = 100 μm. (**C**) The Annexin V assay of seven melanoma cell lines and one human primary melanocyte culture post indicated treatments. (**D**) Protein lysates were prepared under the same treatment conditions as above and were probed with an antibody recognizing full length and cleaved PARP. * indicates *p* < 0.05; ** indicates *p* < 0.01; *** indicates *p* < 0.001. All treatment time were for 48 hours.

The combination significantly (*p* < 0.05 or less) reduced cell viability compared with DMSO or with single drug treated conditions in multiple cell lines, in both BRAF mutated (A375, 1205Lu, SK-MEL 28, 451Lu and WM239a), or NRAS mutated (WM852c) cells (Figure 1A). However, neither drug alone or in combination had a significant effect on normal melanocytes.

Visually, the combination resulted in a more rounded morphology or complete detachment from the plates relative to the single drug treatments or control (Figure 1B), suggesting that the combination induced killing. Annexin V assays demonstrated that the combination dramatically increased apoptosis compared to DMSO or single drug treatment conditions for all seven melanoma cell lines tested (*p* < 0.05 or less) irrespective of the mutation status, but not for the melanocytes (Figure 1C).

Additionally, we analyzed protein lysates from these treatments for cleavage of PARP (Poly ADP-ribose polymerase 1) that is a well-known marker of cells undergoing apoptosis [38]. The combination treatment resulted in the highest level of PARP cleavage relative to other treatments. This was again consistent for all the melanoma cell lines tested irrespective of the mutation status of BRAF or NRAS (Figure 1D). Taken together, these results indicate that the ABT-737 plus GSI-I combination has an increased killing efficacy in melanoma.

### The combination killed the MICs in multiple melanoma cell lines

The sphere formation assay is one of the best *in vitro* methods to study CSCs [39] (Supplementary Figure S1). Melanoma-spheres can be used as a tool to enrich the MICs and to test the potency of drugs [18, 19, 39, 40]. The ALDH (an intracellular MIC marker) assay is another surface-marker independent standard method used to detect MICs [15, 41]. We used both assays to examine the effects of the ABT-737 and GSI-I combination treatment on MICs.

**Figure 2:**
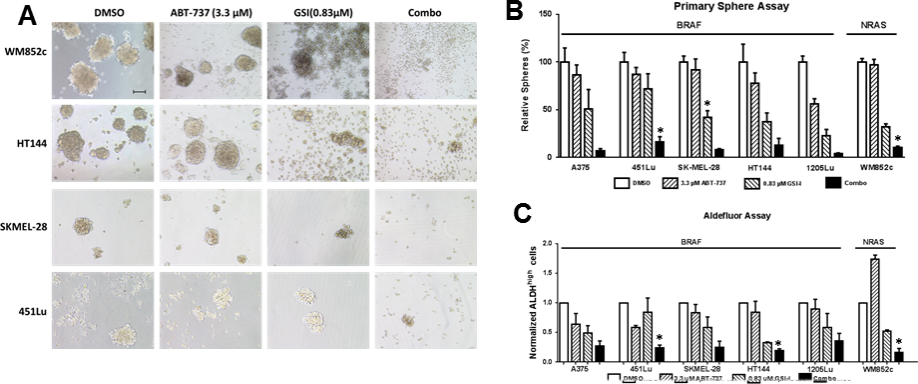
GSI-I combined with ABT-737 killed the MIC population of melanoma cells regardless of the mutation status Melanoma cells were subjected to sphere assay. Spheres were treated with indicated compounds either by itself or in combination for 48 hours, and were then subjected to (**A**) Bright field analysis, Scale bar = 100 μm; (**B**) Quantification of the number of primary spheres; and (**C**) Quantification of ALDH assay. For clarity, we have only marked those cell lines, where the combination is significantly different from DMSO as well as both single drug treatments. * indicates *p* < 0.05.

The combination was better than either of the single drugs in disrupting the primary spheres (Figure 2A and 2B). In all six melanoma cell lines tested, the combination severely disrupted the primary spheres compared to the DMSO (*p* < 0.01) and ABT-737 (*p* < 0.05) single drug conditions, Figure 2A and 2B). The combination also significantly decreased the number of primary spheres compared with GSI-I alone (*p* < 0.001) (Figure 2B) in three out of six cell lines tested. GSI-I by itself significantly decreased the primary sphere in only three out of six cell lines compared to DMSO (*p* < 0.05) and ABT-737 (*p* < 0.01) (Figure 2A and 2B).

In all six melanoma cell lines that we tested, the combination treatment significantly decreased the percentage of ALDH^high^ cells compared with the DMSO control (*p* < 0.01 or less) (Figure 2C). For example, in A375 the percentage of ALDH^high^ cells was 3.6% in DMSO, and the combination treatment reduced the percentage to 1%. In three out of six melanoma cell lines, the combination treatment significantly decreased the percentage of ALDH^high^ cells compared to either the ABT-737 or GSI-I single drug conditions (*p* < 0.05). On the other hand, GSI-I decreased the percentage of ALDH^high^ cells compared with DMSO in only two cell lines (*p* < 0.05 or less). ABT-737 did not significantly decrease the percentage of ALDH^high^cells compared with DMSO in any of the cell lines. Together, this data suggests that the combination treatment is more capable than single drugs in killing the MICs irrespective of the mutational status of BRAF or NRAS.

### The combination inhibited the self-renewability of MICs in multiple melanoma cell lines

**Figure 3:**
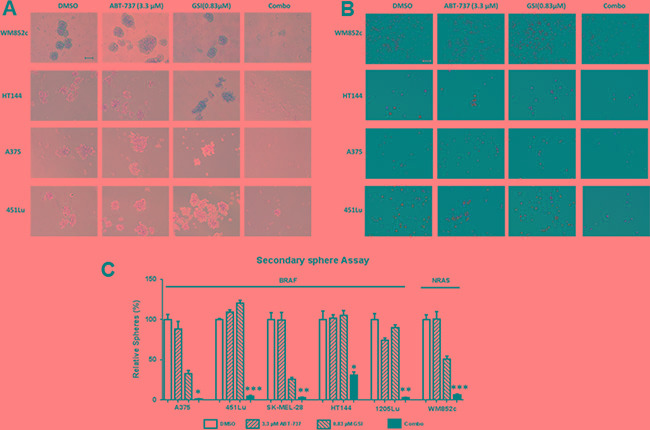
GSI-I combined with ABT-737 inhibited the self-renewability of MICs Secondary sphere assays were performed with the indicated melanoma cell lines. (**A**) Bright-field images of secondary spheres. Scale bar = 100 μm (**B**) Visualization of the secondary sphere cells with the Ethidium Bromide/Acridine Orange (EtBr/AO). Scale bar = 100 μm. (**C**) Quantification of the number of secondary spheres. * indicates *p* < 0.05; ** indicates *p* < 0.01; *** indicates *p* < 0.001. For clarity, we have only marked those cell lines, where the combination is significantly different from DMSO as well as both single drug treatments.

The self-renewal capacities of CSCs contribute to cancer relapse [42]. The secondary sphere formation assays measure the renewability of these cells *in vitro*, thus addressing the vital question of whether the self-renewability of the MICs are altered by the combination treatment (Supplementary Figure S1). The combination treatment almost eliminated all secondary sphere formation (Figure 3A) compared with the DMSO, ABT-737, or GSI-I single drug treatments (*p* < 0.05 or less) in all six cell lines tested, irrespective of their mutation status for BRAF or NRAS (Figure 3C). Thus, these results suggest that the combination may decrease MIC's self-renewal capability.

Visualization of the cells in the above assay using Ethidium Bromide/Acridine Orange (EtBr/AO) staining indicated that majority of cells in the control or single drug treated conditions were alive, but the majority of cells in the combination treatment conditions were dead (Figure 3B).

### The combination increased the NOXA: MCL-1 ratio in both monolayer and sphere conditions

**Figure 4:**
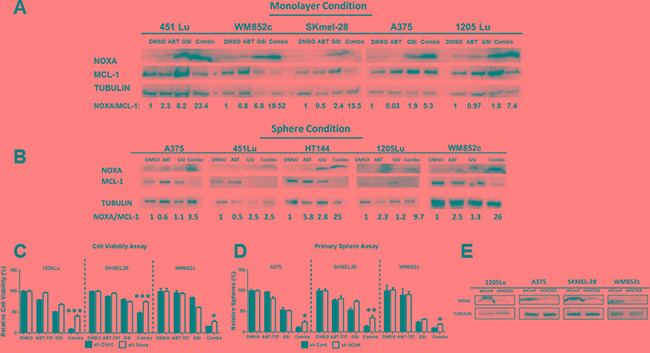
GSI-I combined with ABT-737 increases the NOXA/MCL-1 protein ratio and the increase in combination-induced cell death is partially NOXA -dependent in both monolayer and sphere conditions (**A**) and (**B**) Protein lysates were prepared under the same treatment conditions as in Figure 1 (for monolayer, Figure 4A) and Figure 2 (for spheres, Figure 4B) before being subjected to immunoblot. (**C**) MTS or ATP assays of stable melanoma cells, 1205Lu, SK-MEL-28, and WM852c, carrying a control shRNA (shcontrol) or a shRNA against NOXA (sh NOXA). The viability of the DMSO control for each cell line was set to 100%. The data of 1205Lu and SKMEL-28 cell lines are from MTS assay while WM852c cell line is from ATP assay. (**D**) Primary sphere assays of stable melanoma cells, A375, SK-MEL-28, and WM852c, carrying a control shRNA (shcontrol) or a shRNA against NOXA (sh NOXA). For all the experiments above, cells were treated for 48 hours in monolayer condition or sphere conditions with the following treatments: vehicle (DMSO), 3.3μM ABT-737, 0.83μM GSI-I, or combination of the both drugs. (**E**) Immunoblot confirmed the knockdown of NOXA in the indicated cell lines. * indicates *p* < 0.05; ** indicates *p* < 0.01; *** indicates *p* < 0.001.

GSI-I has been reported to induce NOXA expression specifically in melanoma, breast cancer, and multiple myeloma [32, 43–46], and one of NOXA's main functions is to bind to and inhibit MCL-1 [47]. We therefore tested whether NOXA and MCL-1 are involved in the molecular mechanism of the combination induced killing observed here (Figure 4A). In monolayer culture, GSI-I alone substantially increased the ratio of NOXA/MCL-1 from 2 to 8 fold in all the cell lines tested. However, the combination increased the ratio anywhere from about 5 fold to 23 fold depending on the cell line (Figure 4A). Similar effects from the combination treatment were found in the sphere cultures (Figure 4B). Thus, this shift in the apoptotic protein equilibrium upon treatment is the most likely mechanism for the disruption of the melanoma spheres in our experiments.

### The upregulation of the pro-apoptotic protein NOXA was a significant contributor to the combination-induced cell death

To understand whether NOXA plays a direct role in the combination induced killings, we generated stable NOXA knockdown (KD) lines using a shRNA-mediated approach. KD of NOXA significantly (*p* < 0.05) protected the cells against the combination-induced decrease of cell viability (monolayer condition) (*p* < 0.05) and disruption of spheres (MIC) (*p* < 0.05) for all three cell lines (Figure 4C and 4D). Immunoblot analysis confirmed that NOXA expression was decreased by ~ 70% for all the cell lines tested (Figure 4E). Taken together, the data suggests that NOXA is an important mediator for the killing induced by the combination of ABT-737 plus GSI-I.

### The combination was effective in de-bulking and killing the MICs in relapsed patient samples

**Figure 5:**
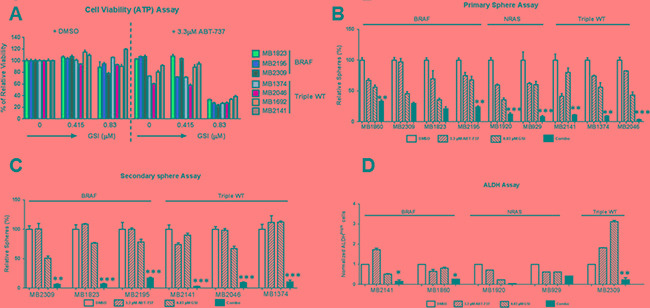
GSI-I combined with ABT-737 de-bulks and eliminates MICs in multiple relapsed patient samples (**A**) ATP assays of seven relapsed melanoma samples treated with indicated treatments for 48 hours. The viability of the DMSO control for each cell line was set to 100%. (**B**) Quantification of primary spheres of relapsed melanoma samples, 48 hours post treatment with DMSO, ABT-737, GSI-I or Combination. (**C**) Quantification of secondary spheres of relapsed melanoma samples. (**D**) Quantification of ALDH assay of relapsed melanoma samples. * indicates *p* < 0.05; ** indicates *p* < 0.01; *** indicates *p* < 0.001. For clarity, we have only marked those cell lines, where the combination is significantly different from DMSO as well as both single drug treatments in Figure 5B to 5D.

To make this study more relevant to the current clinical settings of melanoma, we tested the effect of the combination on tumor samples obtained from relapsed patients (see Supplementary Table S1 for more details). We found that the combination significantly (*p* < 0.001 or less) reduced cell viability compared with DMSO or either single drug treatment, in all the seven melanoma samples tested. The effect was consistent across the samples, irrespective of the mutational status (Figure 5A). The combination treatment also caused severe disruption in primary sphere formation and significantly reduced the number of primary spheres compared to the DMSO (*p* < 0.01) and ABT-737 (*p* < 0.05) in all nine patient samples tested (Figure 5B). The combination severely disrupted the primary spheres in seven out of nine patient samples tested, compared to all treatments, including DMSO, ABT-737 and GSI-I alone (*p* < 0.05 or less) (Figure 5B). In all six samples, the combination also significantly inhibited the formation of secondary spheres compared with DMSO, ABT-737 or GSI-I treatments alone (*p* < 0.05 or less, Figure 5C) regardless their mutation status. ALDH assays with these patient samples consistently showed that the combination treatment decreased the percentage of ALDH^high^ cells compared to the controls, similar to the effects observed in our melanoma cell lines. Unfortunately, we did not have enough material for all patient samples for a proper statistical analysis. We could do statistical tests only on three samples, and in all of them the combination significantly decreased the percentage of ALDH ^high^ cells compared to the vehicle (DMSO) control, ABT-737 or GSI-I treatments (*p* < 0.05 or less) (Figure 5D). Overall, the data suggests that the combination treatment is effective in de-bulking and killing the MICs of relapsed patient samples irrespective of the mutation status (Figure 5).

### The combination increased the NOXA: MCL-1 ratio of the relapsed patient sample lysates in sphere condition

**Figure 6:**
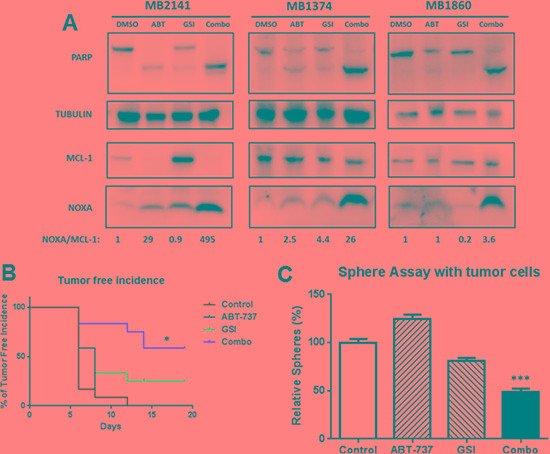
GSI-I combined with ABT-737 increases the NOXA/MCL-1 protein ratio and inhibit the MIC-mediated tumor formation *in vivo* (**A**) Protein lysates were prepared under the same treatment conditions as in Figure 5B and probed for PARP, NOXA, MCL-1and TUBULIN. (**B**) The treatment effects were tested in a xenograft model initiated with a low-number of MIC-enriched cells. For tumor injection, we used the surviving cells from the sphere cultures, upon treatments of GSI-I and ABT-737, either alone or in combination, at the density of 50,000 viable cells per injection. Tumor-free incidence curve shows a significantly longer tumor–free time in the combination group, compared to the vehicle or single drug groups (*p* < 0.05). (**C**) Sphere assays with the tumor cells collected at the end of the animal experiment, and the number of spheres was significantly lower in the combination group compared to control or single drug groups (*p* < 0.001). *N* = 3. * indicates *p* < 0.05; *** indicates *p* < 0.001.

To examine if the mechanism of combination-induced cell death in our patient tumor samples is similar to that in the melanoma cell lines, we performed immunoblot assays for PARP cleavage, NOXA, and MCL- 1. The combination increased the level of PARP cleavage relative to the DMSO or either treatment alone in all three relapsed melanoma patient samples tested, irrespective of the mutation status of BRAF or NRAS (Figure 6A). The combination also increased the NOXA/MCL-1 ratio in all the melanoma patient samples tested (Figure 6A). Thus, this shift in the apoptotic equilibrium is also the likely mechanism for the killing by this combination in our patient samples.

### The combination of GSI-I and ABT-737 delayed MIC-mediated tumor formation *in vivo*

We employed a modified xenograft method with a low cell number implantation to test the differences in tumor initiating ability between the control and treatment groups, as previously described in [27] (Supplementary Figure S2 illustrates the detailed experimental layout). This method makes testing cancer initiation more feasible than the standard series dilution method. Similar approaches have been used previously in assessing tumor initiating ability in other cancer stem cell studies [48–51]. Briefly, we used melanoma cells derived from a relapsed patient sample. These cells were only passaged in the PDX model. We first treated cells *in vitro* with DMSO, single, or combined drugs for 48 hours. We then quantified and implanted 50,000 viable cells from each group, and monitored the tumor growth as readout for the impact of the single/combination treatment on tumor-initiation ability (Supplementary Figure S2).

The mice in the combination group had the longest tumor-free survival time compared to vehicle as well as single drug groups (*P* < 0.05) (Figure 6B). Additionally, the tumor in the combination group was significantly smaller compared to all other groups at day 21 (*P* < 0.05 or less) (Supplementary Figure S3). Tumor incidence rate was calculated as the number of tumors generated /number of implantations expressed as a percentage, and it was significantly lower in the combination group compared with the vehicle or single treatment group (*P* < 0.05) (Figure 6B). To further examine the effects, we performed sphere-forming assays with the single cell suspensions isolated from the surviving tumors at the end of the experiment (Supplementary Figure S2). We found that the combination group exhibited a significant reduction in the number of spheres compared with the drug vehicle or individual treatment groups (*p* < 0.001) (Figure 6C). There was no significant difference between the control and single-drug treated groups in all the above three analyses. Taken together, these results suggest that the combination-treated populations contained fewer MIC-like cells capable of self-renewal.

## DISCUSSION

The current treatments of melanoma include BRAF and MEK inhibitors, or immunotherapy drugs. However, both strategies have limitations of relapse and are not useful for patients that are BRAF-WT or triple-WT. The main findings of this study are 1) Upregulation of NOXA along with inhibition of BCL-2 is a promising approach to kill the bulk of melanoma cells and the MICs. 2) This strategy is effective not only against cell lines, but also against melanoma cells of patients relapsed from current treatments, and against BRAF-WT or triple-WT melanomas for which there are fewer treatment options. 3) These effects in patient samples were also irrespective of the mutation status and the relapsed state.

Relapse is a common challenge for various cancers including melanoma and is explained by the CSC hypothesis. Conventional cancer treatments target the “bulk,” non-CSC tumor cells, leaving behind drug-resistant CSCs. Recently, the cancer treatment paradigm has changed to focus on de-bulking and eliminating CSCs to prevent relapse. This approach has been tested in pre-clinical settings for breast cancer and glioblastoma [40, 52, 53]. Various pharmaceutical companies have focused on designing drugs to target CSCs, and some of those compounds are in clinical trial for lung cancer, ovarian cancer, and solid tumors. Phase I clinical trials are being conducted for eliminating glioblastoma stem cells. MICs are thought to contribute to melanoma's relapse and resistance to treatment [14, 28, 54, 55]. Thus, therapeutically targeting MICs can be a reasonable approach in melanoma to overcome resistance and to prevent relapse. This study tested the effects of the combination treatments of GSI-I and ABT-273 on de-bulking and killing MICs for melanoma treatment. Multiple assays indicated this combination can kill both the bulk and the MICs of the melanoma cells.

Various studies including ours have suggested that targeting BCL-2 family members can be a useful strategy to kill the MICs [24, 27, 56]. Multiple labs including our own have repeatedly shown that NOXA/MCL-1/BCL-2 apoptotic node is a common vulnerable spot for targeting the heterogeneous cell population of melanoma. One of the studies [24] indicate that combining ABT-737 with 4-HPR (an indirect MCL-1 inhibitor) is effective in killing both the bulk of melanoma cells and MICs, but not normal melanocytes. The combination increased NOXA expression and caspase-dependent MCL-1 degradation. This study further validates the above findings that altering NOXA-MCL-1 ratio can be an alternative treatment options for melanoma.

In melanoma, GSI-I treatment has been reported to induce NOXA, in a p53-independent manner and causes mitochondria-mediated apoptosis [32]. Our results also support that GSI-I acts as NOXA inducer in melanoma. We found that GSI-I by itself induces NOXA in monolayer and in some of the sphere-conditions, for both cell lines and patient samples. In addition, knock-down of NOXA protected against combination-induced reduction in cell viability and sphere disruption (Figure 4C and 4D). These results suggest that combination induced cell death is NOXA-dependent.

Although knocking down NOXA protected cells from the combination, it did not completely abrogate the killing effects (Figure 4C and 4D). This may be due to the incomplete knock down of NOXA expression. It is also possible that other proteins may contribute to the killing effects of these drugs. Apart from NOXA, BIM is another pro-apoptotic BCL-2 family member which binds to and abolishes MCL-1's functions [57], and may be important for the effect of certain combination treatments with ABT-737 [24, 27, 58, 59]. TRAIL-induced MCL-1 inhibition leads to BIM-mediated apoptosis [60] and low levels of BIM contribute to melanoma's resistance to BRAF inhibitors [29, 61, 62]. In addition, NOXA-MCL-1-BIM interplay is important for apoptosis induced by drugs such as microtubule-targeting agents [63]. Moreover, BIM can be a downstream molecule of PD-1 signaling in T cells and a predictive T-cell biomarker for response to anti-PD-1 therapy in metastatic melanomas [64]. Thus, we examined the potential roles of BIM in our study. Knocking out BIM, with the CRISPR/Cas 9 technique, did not consistently protect melanoma cells against the combination-induced sphere disruption (Supplementary Figure S4). There was a trend for protection but it was not statistically significant (Supplementary Figure S4). Additionally, we also examined the BH3 only protein, BID. However, we did not detect any protection from knocking down BID (Supplementary Figure S5). These data suggest that the combination of ABT-737 plus GSI-I can induce cell death independent of BIM and BID expression in some situations. The results here are consistent with a recent finding that BIM, BID or PUMA are not essential for activating apoptotic pathways, if the anti-apoptotic BCL-2 proteins are neutralized [65].

We have performed cell cycle analyses in some cell lines. The combination caused a decrease in the percentage of cells in G1 phase and increased the percentage of cells in G2M and S phase (Supplementary Figure S6). However, we have focused our study on the cell death since the targets of these drugs (BCL-2 family members) mainly play roles in regulating cell death.

Studies with different types of GSIs have revealed differences among their mechanisms of action. GSI-I has been found to act like proteasome inhibitors such as bortezomib and MG-132 in multiple cancers [66–71]. On the hand, GSI-IX, GSI-XXI, GSI-XII and R04929097 are specific inhibitors of Notch-signaling pathway [72–77]. The results here are consistent with GSI-I acting as a proteasome inhibitor. Although proteasome inhibitors act in various cancers by inhibiting NF-kB signaling pathway [78–80], in melanoma proteasome inhibitors mediate killing mainly through induction of NOXA [32, 81]. We have previously evaluated the efficacy of killing melanoma cells with proteasome inhibitors (MG-132 and Bortezomib) in combination with ABT-737 [26, 82], and found that both proteasome inhibitors induced NOXA expression and synergistically killed the bulk of melanoma cells by neutralizing MCL-1's function. In both studies, knockdown of NOXA protected the cells from cytotoxicity induced by the combination treatment [26, 82]. Further, other studies have also shown that GSI-I acts like a proteasome inhibitor and induced NOXA-mediated killing [67, 68, 83–86]. Moreover, we found that GSI-I treatment increased the expression of polyubiquitin proteins as further support that it acts as a proteasome inhibitor in melanoma samples (Supplementary Figure S7). Taken together, these results in combination with previous studies suggest that this proteasome inhibitor property of GSI-I is the main cause of the induction of NOXA and the observed efficacy in the combination setting.

To be thorough, however, we also tested whether GSI-I inhibits NOTCH signaling in melanoma. Using immunoblot we probed for the downstream Notch pathway targets HES-5, HES-1, Cyclin D1 and NICD, as others have done [87–89]. NICD was not detectable in the majority of the melanoma samples. For the other three proteins, we did not see any consistent changes in the expression across the majority of the melanoma samples tested (Supplementary Figure S8), indicating that GSI-I is not a specific Notch inhibitor in melanoma.

In summary, we have demonstrated that the combination of GSI-I with ABT-737 killed the bulk and the MICs of multiple melanoma samples, irrespective of the mutational status of BRAF, NRAS or NF-1. It is equally effective against melanoma cells from patients relapsed from current treatments. Our data here suggests that using a combination therapy to inhibit BCL-2 and increase NOXA can be a promising approach to more thoroughly eradicate melanoma. This approach may overcome melanoma's resistance to current treatments. One of the main hurdles in using GSI-I for clinical therapy is the potential side effects such as gastrointestinal toxicity [90–92]. Thus it is important to use low doses of the compound to limit potential off-target cytotoxicity in clinic in the future [20, 92].

## MATERIALS AND METHODS

### Reagents

GSI-I (98.3% purity) was purchased from Calbiochem (La Jolla, CA) or from VDM biochemicals (Bedford, OH) (> 95% purity). ABT-737 (>99% purity) was purchased from Selleck Chemicals. (Houston, TX). GSI-I was used at a concentration of 0.83 μM while ABT-737 was used at a concentration of 3.3 μM for all the assays unless otherwise mentioned.

### Cell lines and patient samples: mutation status and drug treatment

A375, 1205Lu, SK-MEL 28, HT144 and 451Lu lines each have the *BRAF*^V600E^ mutation. WM239a has *BRAF*^V600D^, and WM852c, SK-MEL-2, Hs852T has *NRAS*^Q61R^ mutation.

Patient samples were derived from melanoma biopsy samples of patients relapsed from various treatments (see Supplementary Table S1 for more details): Immunotherapy (MB2141) or BRAF inhibitors (MB1823) or BRAF and MEK inhibitors (MB2309) or multiple drugs/radiation/surgery (MB1692, MB2046, MB1374). The patient samples either harbored a BRAF mutation (MB2309, MB2195 and MB1823), NRAS-mutation (MB1920 and MB929), or were Triple-WT (wild type for BRAF, NRAS and NF1) (MB2141, MB2046, MB1692 and MB1374). These melanoma cultures were validated by the University of Colorado skin cancer biodepository with Melanoma Triple Cocktail staining described in [27].

Other methods including all assays are performed as described in our previous publications [24, 27, 93, 94]. Supplementary Figure S1 provides a schematic of the experimental layout for the primary and secondary sphere assays. At least three repeats of both the primary and secondary sphere assays were done for each cell line/tumor sample. Drug treatment started on day 5 after seeding for primary sphere assays and 24 hours after seeding for monolayer assays (MTS, ATP, Annexin, Cell cycle or ALDH). Drug treatment time was 48 hours for all assays.

### Immunoblot

All cells, both floating and adherent, were collected and lysed using 1X laemmli buffer (Bio-Rad, Hercules, CA). Samples were used in the standard western blot analysis protocol as described previously [25, 95]. The following antibodies were used at suggested dilutions from the manufacturers: PARP, UBIQUITIN and α/β TUBULIN were from Cell Signaling Technology (Danvers, MA); NOXA was from EMD Biosciences, Inc. (San Diego, CA); MCL-1 was from BD Biosciences (San Jose, CA); and HRP-conjugated goat anti-mouse and anti-rabbit antibodies were from Jackson Immuno-Research (West Grove, PA).

### Measurement of cellular viability, cell death, ALDH activity and cell cycle analysis

Cell viability was measured and quantified by using MTS assay or ATP assay (Promega Corp., Madison, WI).

Annexin V-FITC Apoptosis Detection Kit (BD Biosciences, San Jose, CA) was used to quantify apoptosis by flow cytometry in collaboration with the University of Colorado Cancer Center Flow Cytometry Core, according to the manufacturer's protocol. The staining with FITC Annexin V was conducted in conjunction with the vital dye propidium iodide (PI) and can differentiate early and late apoptotic cells.

The Aldefluor kit (Stem Cell Technologies, Vancouver, Canada, #01700) was used to detect the ALDH activity. All the assays are previously described in [96]. All the experiments were repeated thrice for each cell line. For ALDH assay, the data was normalized as the relative fold to visualize the change in the percentage of ALDH positive cells relative to the vehicle (DMSO) control, with the ALDH^high^ cells in the vehicle control (DMSO) condition set as “1”.

Cell cycle analyses were performed using Krishan stain and the data was analyzed by Flow cytometer using ModFit software.

### Creation of short hairpin RNA transduced cell lines

Stable cell lines were constructed as previously described using shRNA Lentiviral Particles from Santa Cruz Biotechnology (Santa Cruz, CA) according to the manufacturer's instructions [25].

### Creation of CRISPR mediated cell lines

BCL-2 family member BIM was knocked out by CRISPR /Cas9 technology. The protocol was followed from [97]. Briefly, the cells were first subjected to Cas-9 lentiviral transduction and then selected for Blasticydin resistance for 5 days. The Blasticydin-resistance Cas-9 transduced cell lines were then subjected to BIM gRNA lentiviral transduction. Functional Genomics Core at UC Boulder provided CRISPR/Cas9 related vectors, which were provided by Dr. Feng Zhang lab (The Broad Institute and the McGovern Institute of Brain Research at the Massachusetts Institute of Technology) [98]. Two different gRNA sequences of the lenti-guide puro-vectors are GCCCAAGAGTTGCGGCGTAT and CAACCACTATCTCAGTGCAA. After transduction, cells were selected with puromycin so that only cells transduced with a stable construct are preserved. The cells were then seeded in 96-well plate at the density of 1 cell/well using MoFlo XDP100 Cell sorter by the University of Colorado Cancer Center Flow Cytometry Core. The single cells were maintained for clonal expansion and each of the clones were expanded and tested to select for the complete knock-out, and screened and verified by immunoblotting of cell lysates.

### Statistical analysis

All the graphs for MTS assay, ATP assay, Annexin assay, sphere-forming assays and ALDH activity assays were created using GraphPad Prism 5 software. Statistical analyses were conducted using the GraphPad Prism 5 software. Specifically, One-Way Analysis of Variance (ANOVA) was used to evaluate if there were any statistically significant differences among all the conditions within each experiment. Tukey post-hoc test was then performed to determine which comparison among the conditions was statistic significantly different. The analyses with *p value* of 0.05 and below were considered significant.

For animal experiments, the survival curve is plotted as the percentage of tumor free incidence on the indicated days and we used Log rank (Mantel-Cox) test for tumor incidence with Graphpad Prism 6 software. Statistical Analysis for tumor volume data was conducted using a 2-way ANOVA followed by Tukey's post-hoc test for pairwise comparisons of mean fold change in tumor volume between treatment groups using Graphpad Prism 6 software. A *p-value* less than 0.05 was considered significant.

### MIC-mediated tumor xenograft studies

Female NCRNU nude mice, aged 5 weeks, were used for the study. All animal experiments are approved by the Institutional Animal Care and Use Committee (IACUC) of the University of Colorado Denver (protocol number 88512(11)1E). We employed a modified xenograft method with a low cell number implantation to test the differences in tumor initiating ability between the control and treatment groups, as previously described [27]. We treated tumor cells from a relapsed patient with DMSO, GSI-I (0.83 μM), ABT-737 (3.3 μM) or combined drugs in sphere enriched condition *in vitro* for 48 hours. After the treatment, spheres were dissociated into the single cell suspension, quantified and 50,000 viable cells were injected in each flank of nude mice and tumors were allowed to grow, with total 12 replicates per group. We then monitored the tumor growth as readout for the impact of the single/combination treatment on tumor-initiation ability. The animals were not treated with any drugs. Supplementary Figure S2 provides a schematic experimental layout of the mouse study. Tumor incidence was determined when the tumor was palpable and about 75 mm^3^ in size. Mice were sacrificed at the end of the experiment and tumors were collected and dissociated into single cell suspension to perform sphere assays.

## SUPPLEMENTARY MATERIALS FIGURES AND TABLES


