# Supplementary material for: Combining a GSI and BCL-2 inhibitor to overcome melanoma's resistance to current treatments
Source: Oncotarget. 2016 Nov 5;7(51):84594–607. doi: 10.18632/oncotarget.13141 (PMC5356684; doi:10.18632/oncotarget.13141)
Supplement: Supplementary file 1 [file oncotarget-07-84594-s001.pdf]

Combining a GSI and BCL-2 inhibitor to overcome melanoma's resistance to current treatments

Supplementary Materials

Supplementary Table S1: Melanoma patient sample information

Sample ID	Mutation status	Treatments before sample collection (Length of treatment)	Histopathological features	Sample site	Duration between treatment and relapse/sample collection
**MB1823**	BRAF V600K	BRAF inhibitor (1 year 8 months)	Nodular	Small Bowel	2.5 months
**MB2195**	BRAF V600E	None	Superficially spreading	Brain	Not applicable
**MB2309**	BRAF V600E	BRAF-MEK inhibitor combination 1 (1 year 5 months) Different BRAF-MEK inhibitor combination (1 month)	Superficially spreading	Distant Subcutaneous	< 1 month
**MB1374**	Triple WT*	Chemo-Abraxene (< 1 month)	Unknown Primary	Distant lymph node	During treatment
**MB2046**	Triple WT*	Surgery	Superficially spreading	Subcutaneous	1 yr. 4 months
**MB1692**	Triple WT*	Surgery	Superficially spreading	Subcutaneous	Not known
**MB2141**	Triple WT	Immunotherapy (4 doses)	Mucosal	Distant Subcutaneous	8 months
**MB1920**	NRAS G12S and PIK2CA E545K	Chemotherapy (8 months), BRAF/MEK inhibitor (4 months), Immunotherapy (9 months)	Unknown primary	Lymph node	> 3.5 years
**MB929**	NRAS G13D; BRAF WT	Immunotherapy, chemotherapy (10 months) small molecule inhibitor (1 month)	Superficially spreading	Subcutaneous	1 month

Note: Triple WT*= Wild type for BRAF, NRAS and NF-1.

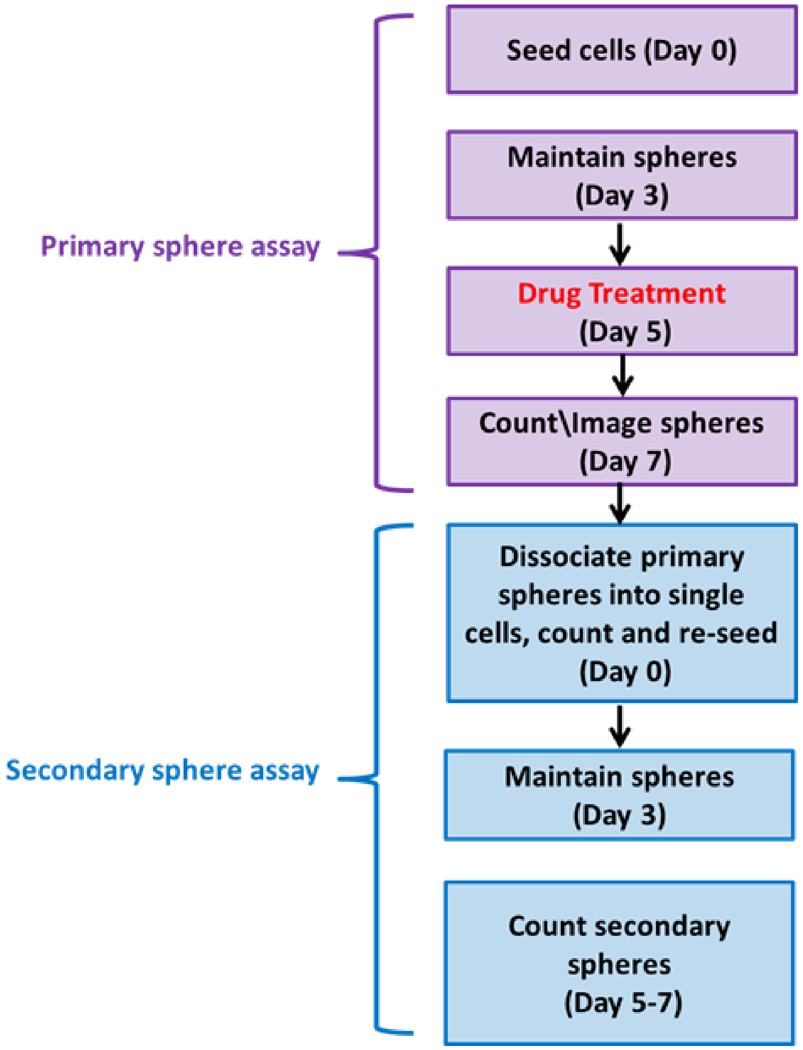

Supplementary Figure S1: Schematics of Primary and Secondary sphere assay.

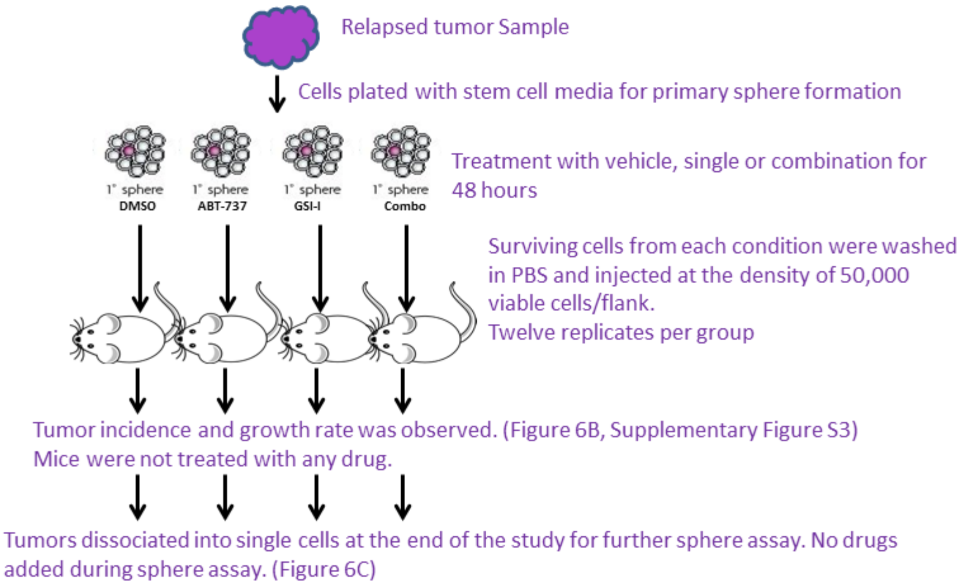

Supplementary Figure S2: Schematic diagram of the MIC mediated mouse xenograft study.

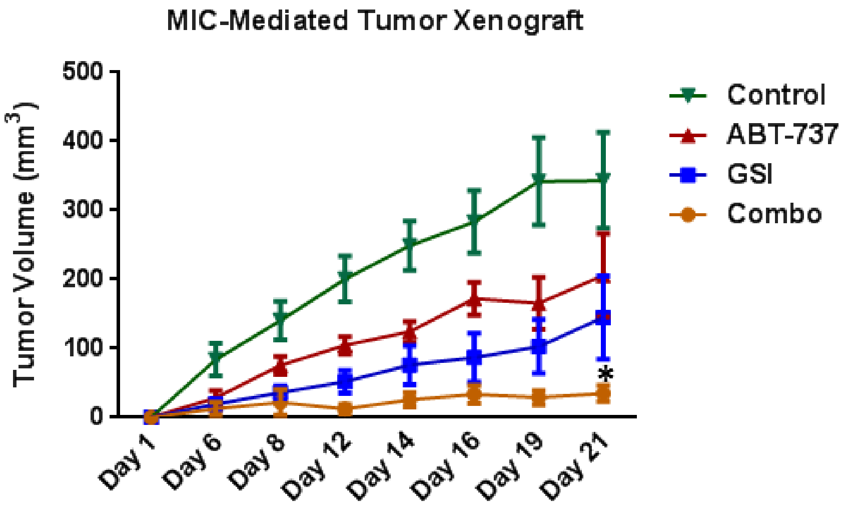

Supplementary Figure S3: Relative tumor volumes in a MIC-mediated mouse xenograft model. The tumor volume at day 0 was set as 100%. The * indicates that the combination significantly delayed the tumor growth compared to Control, ABT-737 and GSI-I on day 21 ($p < 0.05$).

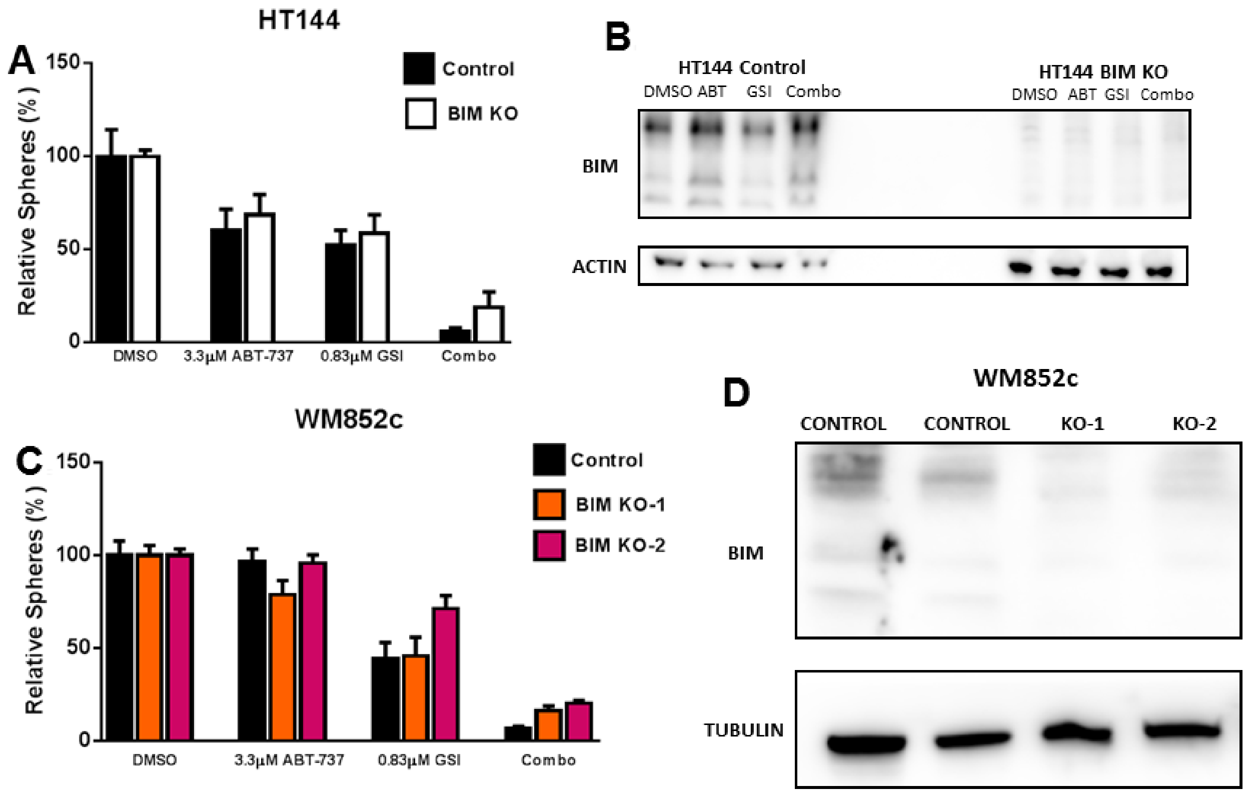

Supplementary Figure S4: HT144 and WM852c melanoma cell lines with BIM knockout (KO) were generated with the CRISPR/Cas9 technology and these cells were used for sphere assays. (**A**) and (**C**) show the quantified sphere assays data with the KO clones of HT144 and WM852c after indicated drug treatments for 48 hours. (**B**) and (**D**) shows immunoblot to confirm KO.

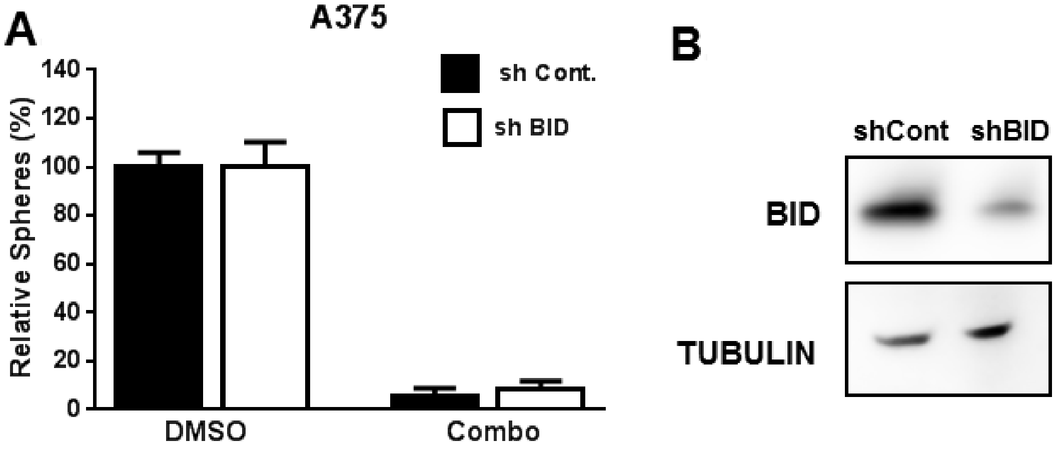

Supplementary Figure S5: (A) Primary sphere assays of A375 melanoma cells, carrying a control shRNA (shcontrol) or an shRNA against BID (sh BID). Cells were treated for 48 hours in sphere conditions with the following treatments: vehicle (DMSO), or combination of the ABT-737 and GSI-I drugs. (**B**) Immunoblot confirmed the knockdown of BID.

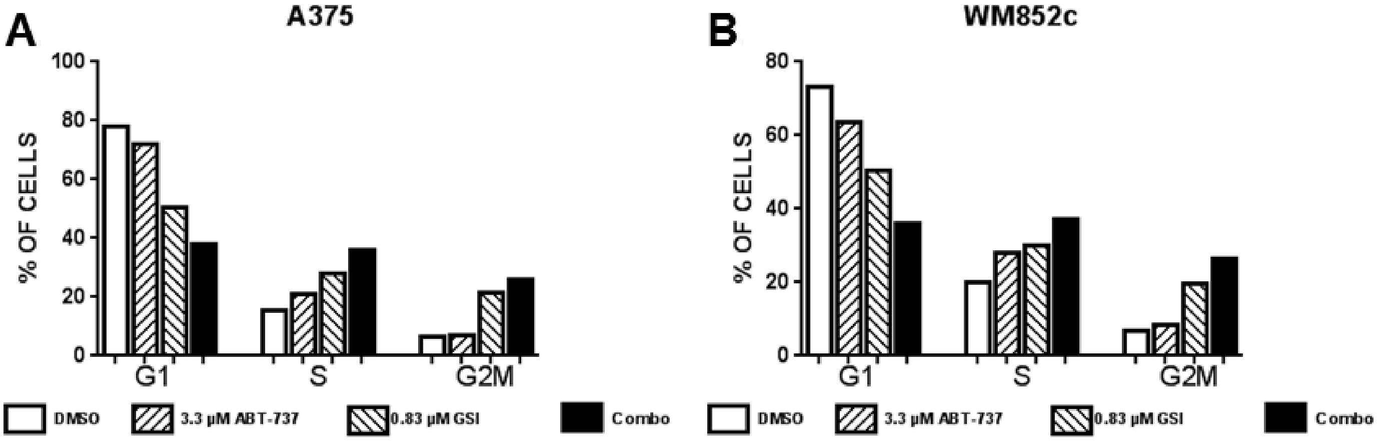

Supplementary Figure S6: Cell cycle analyses was performed in (A) A375 and (B) WM852c melanoma cell lines using Krishan stain and the data was analyzed by Flow cytometry using ModFit software.

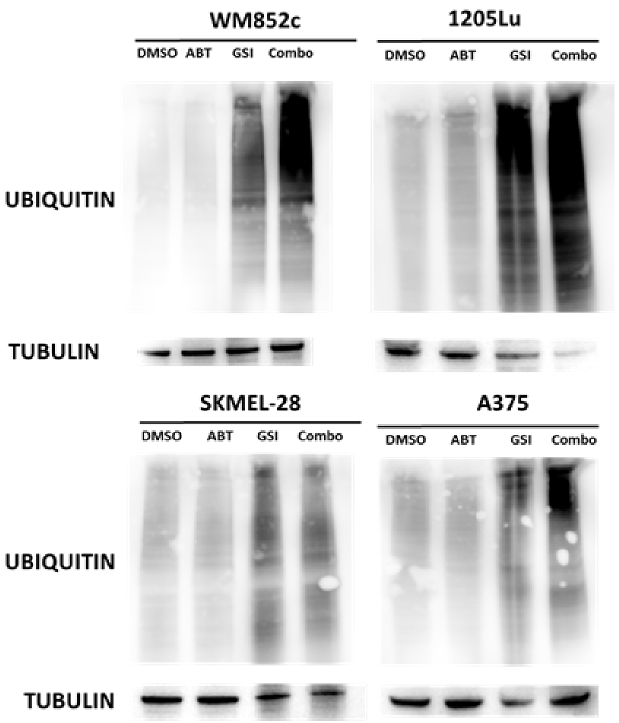

Supplementary Figure S7: GSI-I and the combo increased the expression of polyubiquinated proteins. Protein lysates were prepared after treating the spheres with DMSO, single drug or combination for 24 hours. Blots were probed for UBIQUITIN and TUBULIN.

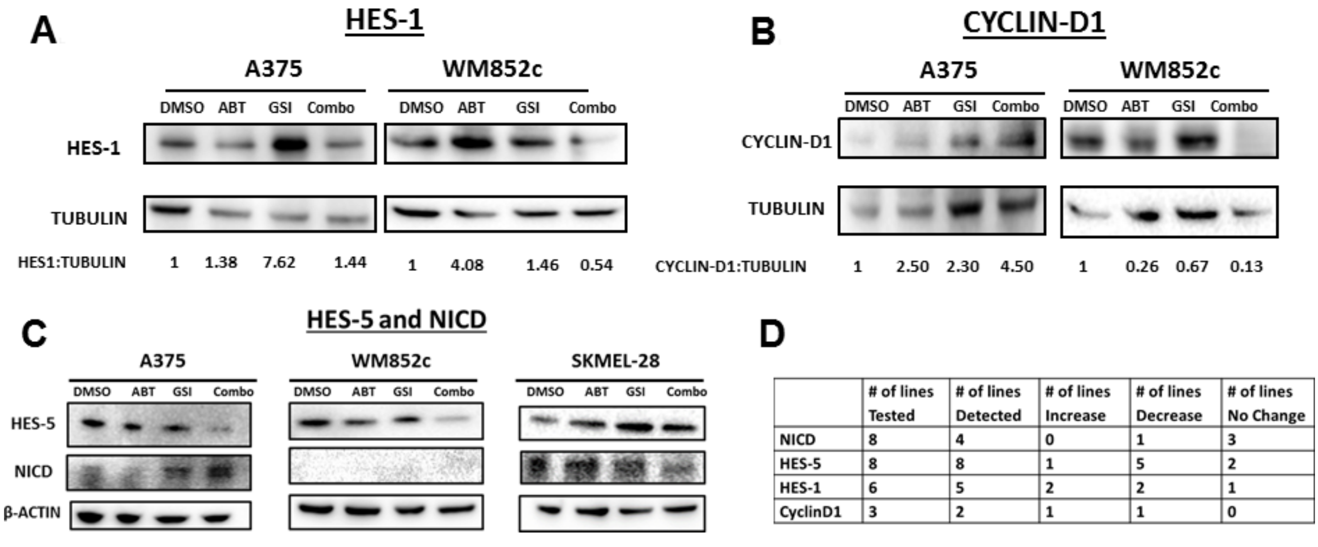

	# of lines Tested	# of lines Detected	# of lines Increase	# of lines Decrease	# of lines No Change
NICD	8	4	0	1	3
HES-5	8	8	1	5	2
HES-1	6	5	2	2	1
CyclinD1	3	2	1	1	0

Supplementary Figure S8: Protein lysates were prepared after 48 hours of treatment in sphere conditions and were tested for Notch downstream targets (A) HES-1. (**B**) CYCLIN-D1 (**C**) HES-5 and NICD. (**D**) Summary of Notch downstream protein response after GSI-I and ABT-737 combination treatment. The numbers indicate the number of cell lines which we tested, detected expression of indicated proteins, and found changes (increase, decrease or no change) in response to combination treatment.
